# Correction: Safety and utility of image-guided research biopsies in relapsed high-grade serous ovarian carcinoma—experience of the BriTROC consortium

**DOI:** 10.1038/s41416-019-0433-6

**Published:** 2019-03-13

**Authors:** T Goranova, D Ennis, A M Piskorz, G Macintyre, L A Lewsley, J Stobo, C Wilson, D Kay, R M Glasspool, M Lockley, E Brockbank, A Montes, A Walther, S Sundar, R Edmondson, G D Hall, A Clamp, C Gourley, M Hall, C Fotopoulou, H Gabra, S Freeman, L Moore, M Jimenez-Linan, J Paul, J D Brenton, I A McNeish, on behalf of the BriTROC investigators

**Affiliations:** 1 Cancer Research UK Cambridge Institute, Cambridge, CB2 0RE UK; 2 Institute of Cancer Sciences, University of Glasgow, Glasgow, G61 1QH UK; 3 Cancer Research UK Clinical Trials Unit, Glasgow, G12 0YN UK; 4 Department of Radiology, Gartnavel General Hospital, Glasgow, G12 0YN UK; 5 Beatson West of Scotland Cancer Centre, Glasgow, G12 0YN UK; 6 Barts Cancer Institute, London, EC1M 6BQ UK; 7 University College Hospital, London, WC1E 6BD UK; 8 Guy’s Hospital, London, SE1 9RT UK; 9 Bristol Haematology and Oncology Centre, Bristol, BS2 8ED UK; 10 City Hospital, Birmingham, B18 7QH UK; 11 St Mary’s Hospital, Manchester, M13 9WL UK; 12 St James Hospital, Leeds, LS9 7TF UK; 13 The Christie Hospital, Manchester, M20 4BX UK; 14 Edinburgh Cancer Research Centre, Edinburgh, EH4 2XR UK; 15 Mount Vernon Cancer Centre, Northwood, HA6 2RN UK; 16 Imperial College, London, W12 0HS UK; 17 Addenbrooke’s Hospital, Cambridge, CB2 0QQ UK

**Correction to:** *British Journal of Cancer* **116**,1294–1301; 10.1038/bjc.2017.86; published online 30 March 2017

This article was originally published under a CC BY NC SA License, but has now been made available under a CC BY 4.0 License.

